# ’One size does not fit all’: a qualitative study of women’s experiences of a self-guided digital intervention, Mamma Mia

**DOI:** 10.1186/s12875-025-03074-8

**Published:** 2025-12-08

**Authors:** Ellen Solstad Olavesen, Silje Marie Haga, Susan Garthus-Niegel, Filip Drozd

**Affiliations:** 1 Oslo Metropolitan University, Oslo, Norway; 2 Regional Centre for Child and Adolescent Mental Health, Eastern and Southern Norway, Oslo, Norway; 3 Institute for Systems Medicine (ISM), Faculty of Medicine, MSH Medical School Hamburg, Hamburg, Germany; 4 Institute and Policlinic of Occupational and Social Medicine, Faculty of Medicine, Technische Universität Dresden, Dresden, Germany; 5 Department of Childhood and Families, Norwegian Institute of Public Health, Oslo, Norway

**Keywords:** Digital intervention, Health promotion, Prevention, Perinatal depressive symptoms, Perinatal period, SWOT, Framework Method, Primary care

## Abstract

**Background:**

The transition to parenthood represents a period with a risk of developing depressive symptoms. Mamma Mia is a universal, self-guided internet-based intervention effective in reducing depressive symptoms and promoting well-being and has the potential to support the transition to motherhood. We aimed to explore how women with elevated depressive symptoms experienced Mamma Mia. The following research questions were addressed: (a) how do women with elevated depressive symptoms experience Mamma Mia, and (b) how can the intervention be improved?

**Methods:**

A descriptive qualitative study was conducted in Norway. Semi-structured interviews based on the SWOT form (Strengths, Weaknesses, Opportunities, and Threats) were conducted with twelve women with depressive symptoms. The data were analyzed via the framework method.

**Results:**

Five overall themes were identified: ‘Usability’, ‘Activities and Learning', ‘One Size Does Not Fit All’, ‘Awareness and Reassurance’, and ‘Seek More Information, Care, and Support’.

**Conclusion:**

The intervention is beneficial, but women with depressive symptoms expressed a need for additional person-centered support in primary care. These findings can guide health personnel in primary and mental health care, in recommending interventions, tailoring support to women in the transition to motherhood, and facilitating help-seeking when additional follow-up is needed.

**Trial registration:**

The Regional Ethics Committee, Norway, approved the protocol as part of the RCT (project number 2012/1716). ISRCTN: RCT no. ISRCTN91808706.

**Supplementary Information:**

The online version contains supplementary material available at 10.1186/s12875-025-03074-8.

## Background

Elevated depressive symptoms during the perinatal period are a public health issue and have negative consequences for the mother, her child, and her partner [26, 31]. However, many individuals with elevated depressive symptoms are less likely to seek treatment and be eligible for clinical services [3, 12]. Mamma Mia was developed as a universal, self-guided internet-based intervention aimed at preventing perinatal depression and promoting well-being [10].

The transition to motherhood is a transformative period, presenting an opportunity for improved well-being but also exposing women to the risk of depression. The global prevalence of clinical depression in the past 12 months in women is estimated to be 6% [39]. Pre-COVID-19, the prevalence of depression in perinatal women ranged from 12 to 17% [30, 41]. During the pandemic, this number may have increased to approximately 26% during pregnancy and 24% postpartum [24, 32]. There is substantial heterogeneity concerning the onset, duration, and severity of depressive symptoms among women. Depression can occur or worsen during the entire perinatal period [1, 35]. Severe symptoms may not necessarily decrease spontaneously, and without identification and early intervention and treatment, these women are likely to suffer a long period of distress, with potential health risks for the mother and the whole family [1]. Compared with nondepressed women, women with perinatal depression reported significantly poorer quality of life. Reduced quality of life, in turn, is related to the persistence and chronicity of depressive symptoms and altered physical and psychosocial functioning [35]. Impairments in psychosocial function among individuals with elevated depressive symptoms are comparable to those among individuals with clinical depression [26]. If left untreated, perinatal depressive symptoms may also have significant and long-lasting consequences, such as reduced quality of life, impaired physical and psychosocial functioning, poor paternal mental health, and potential developmental issues, for the child [7, 31]. These findings indicate that the consequences of impaired maternal mental health are not limited to the children and partners of clinically depressed mothers.

The problem is that perinatal depression is both underdiagnosed and undertreated [7, 12]. Most women who experience perinatal depression do not seek help (or even realize that they are suffering from depression). Both perinatal women and people in general lack knowledge about symptoms, and recognition can be difficult, as symptoms may overlap with those of pregnancy and the postpartum period [6]. Furthermore, negative or trivializing beliefs about perinatal depression are common [6]. Individual factors such as shame and perceived stigma are common barriers to seeking help, as are structural factors such as restricted service accessibility, limited resources, and a lack of coordination between perinatal and mental health professionals [6, 7, 20]. Depression and elevated depressive symptoms are significant public health issues due to their prevalence and because individuals with elevated depressive symptoms are less likely to seek treatment and be eligible for clinical services, as they may not be detected through routine screening assessments [3, 7].

The World Health Organization recommends evidence-based approaches to promote well-being, prevent mental health problems, and treat perinatal depression [40]. Internet-based self-help programs that are universally delivered (i.e., provided to all perinatal women), regardless of risk or symptom level, hold promise owing to their accessibility and flexibility. Digital education is beneficial for improving knowledge and clinical outcomes and increasing self-efficacy, preparedness, and confidence [29]. The effectiveness of psychological interventions is greater among depressed women and primiparas, as they require more emotional and educational support than multiparas do [25]. Moreover, mobile technologies are potentially valuable tools for self-care, early recognition, and reduction of depressive symptoms, extending care beyond health services [4, 23]. A meta-ethnographic review of qualitative studies revealed that digital technologies for the perinatal period increased awareness, knowledge, empowerment, and self-confidence. Furthermore, digital interventions can normalize experiences of emotional distress and are beneficial in terms of the features of digital platforms [23]. Despite the effectiveness and acceptability of digital interventions, this mode of treatment delivery is not always preferable. According to a survey, only 26% of adults preferred self-guided digital treatment as an option for psychotherapy [6]. A meta-ethnographic study of 27 qualitative studies on digital technologies emphasized the need for extended support, diverse topics, real-time feedback, and nonjudgmental professional advice concerning digital interventions [23].

High attrition and low adherence are also common challenges in internet-based interventions, and typical barriers are severe mental health issues, technical problems, and a lack of personalization [2]. Mamma Mia has been shown to be effective in preventing depressive symptoms and enhancing well-being. A prior study on Mamma Mia revealed positive changes among users, suggesting the potential for both health promotion and prevention of depression in the perinatal period [9]. In a randomized trial of Mamma Mia, participants presented with fewer depressive symptoms than those in the control group, and a higher initial EPDS score corresponded to a greater effect of Mamma Mia. However, women with elevated depressive symptoms and low socioeconomic status had higher dropout rates [16]. In addition to the randomized trial, Mamma Mia was previously assessed as a user-friendly, high-quality, and credible intervention in a feasibility study [15]. A recent qualitative study including women who either completed or dropped out of the intervention revealed that the educational content promoted awareness and self-reflection as well as attachment to and bonding with the child [34].

Understanding the user experiences alongside usability and effectiveness evaluations of digital interventions is thus crucial. Therefore, we aimed to explore the experiences of Mamma Mia in women with elevated depressive symptoms.

## Methods

### Aims

The primary aim of this study was to investigate how perinatal women with possible depression experience using the Mamma Mia program. The purpose was to identify the strengths and weaknesses of Mamma Mia and generate knowledge to devise recommendations for future improvements. A secondary aim was to investigate factors that may either facilitate or limit the dissemination, uptake, and use of the program. More specifically, potential dissemination strategies and program components or content associated with good or poor program utility/use should be identified. Taken together, these findings may be applied to support the use of the program among prospective perinatal women with elevated depressive symptoms. This study contributes to the literature on internet interventions by providing qualitative accounts of perinatal women with probable depression and their perceptions and experiences with self-guided programs and identifying the needs for such programs among this user group.

### Design

This was a qualitative study in which we used the Strengths, Weaknesses, Opportunities, and Threats (SWOT) format to guide the interviews and to allow for a rich exploration of Norwegian women’s experiences with Mamma Mia during the perinatal period.

### Recruitment and participants

Criterion-based sampling was utilized to extract a sample with elevated depressive symptoms, as measured with the EPDS (≥ 10). The inclusion criteria for participation in the RCT were being a pregnant female aged ≥ 18 years, less than 25 gestational weeks, and having provided a valid e-mail address. The implicit inclusion criterion included reading and understanding Norwegian at the high school level. Women with an EPDS score ≥ 10 at baseline were invited to participate in the present study.

A total of 12 women who scored at or above the cutoff of 10 on the EPDS were selected and consented to participate in interviews. The purpose of this selection process was to explore the experiences of women with depressive symptoms at baseline to ensure diversity in perspectives, as well as shared experiences among the participants. For a description of the characteristics of the participants, see Table 1. The mean age of the participants was 30.75 years (*SD* = 5.9 years, range = 21–38). As shown in Table 1, all participants were married or living with a partner, and four were primiparous. All, except one, were Scandinavian (i.e., Norway, Sweden, or Denmark) and had a college or university degree. By examining the women’s baseline scores, it was clear that they experienced high levels of both positive and negative affect but had higher scores for positive affect than for negative affect. Most women reported slightly below-average satisfaction with life, and all had an EPDS score of 10 or more, indicating experiences of mild to moderate depression.

**Table 1:** Baseline characteristics of the participants

Characteristics	*N* = 12
Age (years), mean (SD)	30.75 (5.9)
Education	
≤ 1–3 years college or university	5 (41.67%)
≥ 1–3 years college or university	7 (58.33%)
First language	
Scandinavian	11 (91.67%)
Non-Scandinavian	1 (8.33%)
No. of previous children	
No previous children	4
≥ 1 children	8
EPDS^a^	10–18 (Mean 13.17)
SWLS^b^	9–24 (Mean 17.17)
PANAS^c^	
Positive affect	22–42 (Mean 29.42)
Negative affect	15–37 (Mean 26.42)

^a^Edinburgh Postnatal Depression Scale

^b^Satisfaction with Life Scale

^c^Positive and Negative Affect Schedule

The start date of Mamma Mia (i.e., the first program day) ranged from 10.03.14–03.11.14. The number of completed sessions in Mamma Mia ranged from 1–44 at the time of the interview. Eight (66.7%) had become mothers at the time of the interview, while 3 (25%) were still pregnant. Before the interview, information about the overall purpose of the study was given, and the participants provided informed consent to be interviewed.

### The intervention

Mamma Mia has been publicly available since 2016 in Norway, and consists of 44 sessions over 11 months. The program starts at gestational week 21 and ends 6 months after birth. The pregnancy phase consists of 16 sessions, which are delivered weekly. The maternity phase consists of 28 sessions with the highest intensity 2–6 weeks postpartum to support women in the early postpartum period. The sessions in the maternity phase are delivered weekly at first and then two and three weeks apart. Each session is designed to take 10–15 min and must be completed before users can access the next session.

Mamma Mia targets risk and protective factors to prevent distress and ease the transition to motherhood. All the sessions include themes specific to the perinatal period, exercises, and techniques, which are delivered by interactive websites in a tunneled design. The key elements in Mamma Mia are fully described by [10] and include (1) assessment of depressive symptoms,(2) metacognitive therapy; (3) positive psychology, such as mindfulness, gratitude, and exercise; (4) couples’ therapy; (5) information on breastfeeding; and (6) psychoeducation.

### Data collection

#### Measures

Demographic information such as age, education, first language, and marital status was obtained at baseline (gestational weeks 17–24). The participants also completed the following measures as they were enrolled in the study:

- The *Edinburgh Postnatal Depression Scale* (EPDS; [5]) is a widely used self-report measure to identify women at risk of perinatal depression both in research and in clinical practice, with good evidence of sensitivity and specificity [14]. The scale consists of 10 items that are used to assess depressive symptoms in the past 7 days. The participants rated items such as “*I have been so unhappy that I have been crying*” and “*Things have been getting on top of me*”. Each answer is given a score from 0 to 3, with a total score ranging from 0 to 30.
- The *Satisfaction with Life Scale* (SWLS; [8]) is a 5-item instrument used to measure global cognitive judgments of satisfaction with life. The participants rated items such as “*I am satisfied with my life*” and “*So far I have gotten the important things I want in life*” on a scale from 1 “*strongly disagree*” to *5 “strongly agree*”.
- The *Positive and Negative Affect Schedule* (PANAS; [36]) is a self-report measure that consists of 20 items that measure positive (e.g., “*attentive*” and “*inspired*”) and negative affect (e.g., “*nervous*” and “*irritable*”). The participants indicated to what degree they had felt this way in the past week on a 5-point scale from 1 “*Not at all*” to “*Very much*”. Scores range from 10–50 for both the positive and negative affect scales, with lower scores representing lower levels of positive or negative affect and higher scores representing higher levels of positive or negative affect.

#### Interviews

Individual semi-structured interviews in the open-ended Strength, Weaknesses, Opportunities, and Threats (SWOT) format were conducted to explore women’s experiences with and perspectives on Mamma Mia [9, 17]. The method allows the researcher to collect both open-ended data and to further explore participants’ thoughts, attitudes, and beliefs about Mamma Mia.

The interviews were carried out between July 2014 and December 2015. Their duration ranged from 48 min to 1 h and 50 min (*M*: 1 h and 16 min; *SD*: 21 min). The interviews were conducted at the Regional Centre for Child and Adolescent Mental Health (RBUP) or by phone with one participant and a researcher per interview. All interviews were conducted in Norwegian, recorded digitally and transcribed verbatim. To ensure confidentiality, all identifying information was removed from the transcripts, and pseudonyms were given in NVivo. The selected quotes were translated from Norwegian to English using Paperpal. To minimize potential bias and avoid misinterpretations, we carefully reviewed the quotes to ensure accurate translation of the meaning and experiences of participants. A bilingual co-worker back translated the quotes to ensure credible and accurate representations of the participants’ views and experiences.

The interviews consisted of four questions emphasizing the SWOT dimensions, where the participant was allowed to speak openly with a minimum of interruptions, rather than probing questions from the interviewer to elaborate on the experience. This was done to minimize the interviewer's impact on the participants’ answers or influence the conversation in a certain direction in which the interviewer is interested. After each interview, the interviewers worked through the SWOTs to clarify participants’ views and allow for any additional perspectives or elaboration of information. The purpose is to obtain a more detailed explanation of the information given and to avoid potential misinterpretations. It also encourages the subject to reflect further on the question at hand.

The questions were as follows:

**Table:** 

Please tell me about what you perceive as the advantages of and what seems to work well with Mamma Mia; (strengths)
Please tell me about what you perceive as the disadvantages of and what does not seem to work well with Mamma Mia? (weaknesses)
Please tell me about what you consider to be the possibilities of improving the quality of Mamma Mia; (opportunities)
Please tell me about what you consider to be the obstacles to Mamma Mia; (threats and challenges with the Mamma Mia program)

The interviews were conducted by three experienced researchers, one of whom was competent in perinatal depression, the second in internet-based intervention, and the third interviewer was a master’s student in psychology.

### Data analysis

Data analysis was carried out via SPSS Statistics (v. 29) to describe the sample. Descriptive statistics were performed by calculating the frequencies, means, and standard deviations of the baseline data. The research team conducted a framework analysis of qualitative interviews via inductive coding. This approach was appropriate, as we aimed to investigate specific issues regarding the experiences and perceptions of Mamma Mia. Initially, the data were coded in vivo to capture the dimensions or ranges of categories and to develop the coding tree in NVivo (v. 11). In the coding process, codes were compared with other codes, with a focus on similarities within and differences between codes. In the process of descriptive coding, similar codes are recognized and grouped into categories [28]. Patterns of underlying meaning across categories were grouped into themes. Comparative reading continued throughout the analysis process as categories and themes took form, and the themes were refined and revised.

Data analysis was carried out by identifying themes for each interview within and across the strengths, weaknesses, opportunities, and threats. The seven stages of framework analysis [13] were followed: 1) transcription, 2) familiarization, 3) coding, 4) developing a working analytical framework, 5) applying the analytical framework, 6) charting data into the framework matrix, and 7) interpreting the data. Contextual notes and the researchers’ reflective journal informed the analysis. The overall objective of framework analysis is to identify, describe, and interpret key patterns within and across cases within the phenomenon of interest. The framework enables comparisons across cases and the identification of similarities and differences [13]. An overview of the framework analysis is available as a supplementary file (Additional File).

The frequency was determined based on the number of participants’ responses within each theme. On the basis of the recommendations of Hill and colleagues [19], the descriptor *generally* consists of data from all or all but one participant (12 or 11), *typical* for more than half of the participants up to the cutoff for general (6–10), and a *variant* for at least two participants up to the cutoff for typical (2–5). Data for just one case that was not consistent with any of the themes were deemed *rare* [19]. We describe only those results that were *general*, *typical,* or *variant* in the results section.

### Rigor

Preunderstanding is important to consider, as this can influence the conclusions that can be drawn. The research group, which included one male and three females, had varying professional backgrounds and experience. Three of the authors are experienced researchers (FD, SMH, and SGN), and four are experienced in perinatal and child healthcare and/or mental health care (FD, SMH, SGN, and ESO). Two of the authors performed the interviews (FD & SMH) and thus had an inside perspective regarding data collection, whereas one author (ESO) took part in the analyses from an outside perspective. ESO prepared the manuscript, while FD reviewed the drafting of the manuscript, and all the authors revised it critically for important intellectual content. All authors gave final approval for the version to be published.

### Ethical considerations

Written consent and permission to contact participants were obtained during recruitment to the RCT. Oral consent was obtained from the participants before the interviews, who were informed of the study's overall purpose and consented to the publication of de-identified data. Further, the study was conducted according to the ethical principles: risk/benefit assessment, confidentiality, and respect for human dignity as stated in the Declaration of Helsinki [38]. The Regional Ethics Committee, Norway, approved the protocol for the randomized trial (project number 2012/1716, ISRCTN91808706, registered 09/12/2013).

## Results

Overall, the strengths (177) and opportunities (110) outweighed the weaknesses (101) and threats (46) in the number of responses. The participants listed several opportunities, which mainly addressed weaknesses or threats as suggestions for improvement. Five major themes and associated subthemes emerged from the framework analysis: ‘Usability’, ‘Knowledge and Self-care’, ‘One size does not fit all’, ‘Awareness and Reassurance’, and ‘Seeking more information, care, and support’. These are described in detail below.

### Usability

This theme captures participants’ experiences with the intervention’s interface, accessibility, and adaptability to daily life. While overall usability was positively received, described as easy to use and user-friendly, participants also identified specific areas for improvement, reflecting a nuanced engagement with the intervention.

*“After all, it is very straightforward and easy to use. I use Mamma Mia on my phone, and an email comes up, so it is very user friendly. I think that each session took a reasonable amount of time.”* (P06, Strength).

Participants appreciated the simplicity of the program and its integration into everyday routines. However, several usability challenges emerged, particularly related to mobile functionality. The lack of a mobile application and limitations when accessing the program on their phone were seen as barriers to seamless use, especially when navigating or completing tasks on smaller screens.

“*And then I have mostly read and listened to the sessions on the train, on my iPhone… Answering all these questions it is a bit clumsy on the iPhone. That could have been an area of improvement. It could be more mobile-friendly.”* (P04, Opportunity)

Suggestions for improvement included the addition of a top-level menu to enhance navigation, options to skip irrelevant content, and greater flexibility in pacing: *“… you have to click through one slide, or page, to move on. I wish sometimes there was a top menu like that where you could just click… Being able to manoeuvre a little bit easier in the program, that’s what I have missed”* (P01, Opportunity).

*“Those movies have not been quite relevant to me then. Therefore, I have just skipped them when I have figured out the topic…It would have to be that you could have a 'Skip this session' button for me.”* (P05, Opportunity)

The frequency and structure of sessions were also expressed as potential stressors. For some the weekly format felt burdensome, contributing to lagging behind and emotional strain. These suggestions reflect a desire for greater autonomy and personalization, enabling users to tailor the experience to their individual needs and capabilities, particularly during the early postpartum period.

*“I wanted longer intervals between each session, not one assignment or one email each week. I would have liked to have at least two weeks in between… Therefore, if it had been possible to be allowed to choose for oneself…”* (P09, Opportunity).

This was especially demanding for those with children and a busy work situation, adding a burden to their daily life and emotional distress.

*“The only thing is, I felt like I was lagging behind, there's a lot of things you were supposed to do all the time, and then you were lagging behind with this as well. That was a truly bad feeling.”* (P09, Weakness).

Overall, these reflections on usability reveal a balance between appreciation for simplicity and a critical awareness of how design features can either support or hinder engagement. Their input suggests that enhancing flexibility, navigation, and developing a mobile application could improve the user experience.

### Knowledge and self-care

This theme captured how participants engaged with the educational and emotional content of the Mamma Mia program, highlighting its role in supporting both cognitive understanding and practical self-care during the transition to parenthood. The theme is divided into two subthemes: *tasks and exercises* and *relevant information*.

*"What has been good about it, has been the small pieces of advice that have been provided. Not all of the film clips, but the small advice regarding both us as a couple and the relationship to the child. It is truly good to have a program that follows [pregnancy and the child's] development."* (P02, Strength)

Participants generally valued the program’s educational content, especially its alignment with their parenting journey. The integration of developmental insights and parenting tips was seen as helpful and accessible. However, some women noted challenges in retaining and applying the information in their daily lives, suggesting a need for more interactive and reflective components, such as journaling prompts or follow-up tasks, to reinforce learning. Some women also suggested providing instructions alongside videos and demonstrations for a more practical learning experience. Practical exercises, such as breathing, meditation, relaxation, and gratitude, were particularly appreciated for their calming effects and valuable for everyday self-care. *“Everyone has good days and bad days. So, if I feel that one day has been truly sad, finding a positive thing during the day made it slightly more positive.”* (P02, Strength).

These tools were described as timely and supportive, especially during periods of stress or emotional overwhelm.

*“I had not put on that much weight, so the midwife was truly worried about it. We spent the whole consultation calling the hospital, just to get an extra ultrasound appointment, and then it was like, yes, then we were done, and then I was ‘Okey, I'm going to give birth soon. It is getting closer, and my maternal leave is approaching fast’, and I just felt like I was full of thoughts and was just like, ‘Oh, my God, now I actually think I'm capitulating, cannot take it anymore now’. Then, that email came in yesterday and the session with the relaxation and breathing exercise and stuff like that, and then I kind of felt ‘Okay, now I can handle it, it is okay somehow’. Therefore, I have truly liked that.”* (P10, Strength)

For some, the program even influenced health behaviors, such as smoking cessation, indicating its potential to promote broader well-being.

*“I'm not sure how it is worked out concerning myself; but it worked out the way that I have stopped smoking during pregnancy, for the baby's sake.”* (P04, Strength)

Overall, this theme underscores the importance of combining educational content with actionable self-care practices and tailoring delivery to meet the diverse needs of perinatal women.

### One size does not fit all

This theme captures the limitations of universal interventions like Mamma Mia, emphasizing the need for greater personalization to accommodate diverse experiences and needs among perinatal women. While the intervention was generally appreciated, participants noted that its content and pacing were not always relevant or sufficient, particularly during periods of emotional difficulty or for women with prior parenting experience. Multiparous women often felt that Mamma Mia repeated information they already knew, yet they identified gaps in support specific to their circumstances, such as guidance on preparing older siblings for a new baby.

“*I have had a challenge with how do you communicate to a two-year-old that ‘there's a baby in my mommy's womb’, right? Probably a lot going on with him. So, a little advice and guidance about that could be perfect, for example.”* (P08, Opportunity).

Participants’ feedback suggest that prior experience does not eliminate the need for tailored support but rather shifts the nature of that support. Practical barriers, such as time constraints, competing responsibilities, and fatigue further complicated engagement with the intervention. These challenges underscore the importance of a flexible, responsive design that can be adapted to users’ life contexts.

*"You're very tired, and if you're going to take care of yourself and take care of a six-year-old, that has been a challenge that I have not received any help with in Mamma Mia.*” (P07, Threat)

Despite these critiques, participants recognized the potential value of Mamma Mia, especially for first-time mothers or those experiencing emotional vulnerability.

“*Because I'm one of those people who probably would have benefited from having that program the first time because I got postpartum depression then. And I was very unprepared.*” (P12 Strength)

Even though participants underscored the limitations of a universal intervention in addressing diverse informational and emotional needs, some expressed concern about differentiating the intervention too much, fearing it might exclude women or making the design overly complex.

*“Perhaps one should have differentiated between those who say they have..., those who feel they are struggling then... There is certainly a greater need for those who need that support as well. But, it is silly to make it different for them, too.*” (P05, Opportunity)

Overall, this theme calls for a more nuanced approach to intervention design and delivery, one that acknowledges varying levels of experience, emotional needs, and life circumstances, and offers adaptable or additional pathways to support.

### Awareness and reassurance

This theme reflects how Mamma Mia supported emotional awareness and provided emotional reassurance during the perinatal period.

*“I think it is good to have Mamma Mia because it reminded me of what I was going through. Even though I kind of knew it... have the time to think, and yes… ‘There is a child in here’. Yes, it is a busy time. Absolutely. To get into wonder and joy during pregnancy. To have a breathing space in everyday life, yes, that is what I think it was.”* (P06, Strength)

Psychoeducational elements, including the EPDS, were effective in prompting self-reflection and normalizing emotional fluctuations. Repeated assessments encouraged participants to monitor their emotional states over time, fostering a sense of continuity and self-awareness:

*“I also think they have been good these questions about how you're doing, whether you're depressed or not. I think that has been good, for me, too. It made me think a little bit about how I'm truly feeling. I have also thought about why I feel that way. And the repetition [the EPDS]. Perhaps in the case of those questions, that one could be reminded of what one answered the last time.”* (P07, Opportunity)

The “parent room” feature was highlighted as a valuable tool for enhancing communication within relationships. It facilitated constructive dialogues between partners, allowing for shared reflection. This contributed to a sense of relational stability and mutual understanding during the perinatal period.

“*We [the participant and partner] got to talk a little bit more about it, about how we do things and then set aside time to talk, and about family things, not just what we have to do this week. A little bit about how we think we're truly doing, to think it through. If there were any situations, that we wished we had dealt with differently. It made it easier to bring things up, issues that are bothering us. Yes, bring subjects up more constructively and calmly, rather than waiting until it boils over.*” (P06, Strength)

The women also expressed appreciation for Mamma Mia’ affirming tone which made them feel seen and accepted in their emotional complexity. Mamma Mia provided reassurance that a range of feelings, including ambivalence, joy, fatigue, were valid and part of the transition to motherhood. This validation appeared to ease the integration of new roles and identities.

*“Yes, I'm not sure how, but it somehow felt positive. I had a greater need to feel like it was still me in all that. That I'm allowed to have all these feelings and be allowed to be myself in it. Because I have not just felt this wonderful family life happening. It is kind of something I have had to grow into. And I have. Through pregnancy. The division [*into rooms*] has probably allowed me to be myself. I have the right to feel what I feel. At the same time, maybe it made me merge a little easier into the role I'm in now.”* (P04, Strength)

In general, this theme addresses emotional literacy and relational support in perinatal interventions. By fostering awareness and offering reassurance, Mamma Mia helped participants navigate the psychological landscape of pregnancy and early parenthood with greater confidence and self-compassion.

### Seek more information, care and support

The final theme highlights participants' need for additional information, care, and support. The main theme covers two subthemes: *support in difficult situations* and *having someone to contact*. While participants appreciated the program’s suggestions for managing emotional and physical challenges, some found help-seeking difficult, especially during periods of distress. Several women indicated a preference for being proactively contacted rather than initiating contact themselves, particularly when scoring high on the EPDS. *“On a different note, I thought when I started the Mamma Mia program, it is going to be good, because if I get depressed and if I feel really bad, I'm going to get identified. However, you will not… So, I do not know if one can somehow get identified if it is truly not going well for one… I'd rather had someone ask me, ‘is it okay for us to call you’?”* (P07, Weakness).

Participants emphasized the importance of connecting with others, professionals, or peers. They stated that connecting with others who have similar experiences can be essential when seeking answers and support during pregnancy. They expressed a desire for face-to-face interactions, opportunities for asking questions, receiving guidance and validation of their emotional experiences. The absence of personalized feedback was seen as a limitation, especially for those with a history of depression or ongoing emotional struggles, and a call for human interaction and follow-up on mental health.

*“I have missed a person to discuss things with, about what the program discusses, I'd rather have a person to discuss with. But it is a internet based self-help program, it’s not a person to talk to. I probably would have needed to discuss how I'm doing, because I have been… depressed for a long time, taking antidepressants. What I needed was someone to discuss it with, and someone who I could tell ‘I am like this and feel that way and I'm so tired’. In addition, someone who could then personally respond to it, and say yes, but that is the way it is. Or say that… Either say that ‘yes, but it is perfectly normal and that is the way it is, and you react perfectly normal like everyone else’, or someone who might say ‘this does not look so good, maybe you're starting to get a little depressed, you should probably follow it up a bit more’. I needed that.”* (P07, Weakness).

Suggestions included integrating the program with health care services, such as GPs, or Maternity and Child Health Care Services, to ensure broader dissemination and more direct support. The participants suggested that health personnel should recommend and disseminate the program:

*“…establish cooperation with either a GP or a health center and the midwifery service. It was a coincidence that I came across Mamma Mia through a website. …there are probably many people who can benefit from it.”* (P04, Opportunity)

They further described how health personnel could provide additional support, which was described mainly as listening, validation, normalization, and guidance.

*“It would be nice if you got some kind of validation… I feel the need to get a professional's opinion on the matter then, kind of say that ‘this indicates that you might be depressed’, etc. That you're supposed to endure what you're going through, etc. Get some kind of confirmation that you feel this way and that, and possibly what you can do about it.”* (P11, Opportunity).

Participants also recommended adding features like discussion forums, personal narratives, and expert-led content to foster a sense of community and normalize emotional challenges in the program, which suggest a need for a more tailored intervention.

*“Videos or texts about personal experiences with postpartum depression, how it feels, and so on... It could be a nice thing to look at... Possibly some forum, a platform with others. In addition, maybe experts, a psychologist.”* (P11, Opportunity)

Overall, this theme underscores the importance of responsive, relational, and accessible support in digital interventions. It calls for a more integrated approach that combines psychoeducation with opportunities for meaningful interaction and personalized care.

## Discussion

This study explored the experiences and perceptions of perinatal women with elevated depressive symptoms who used the internet-based intervention, *Mamma Mia*. Overall, women expressed positive views of the intervention, valuing its ability to provide information, normalize emotional experiences, support self-care, and increase awareness of well-being. Importantly, participants also identified areas for improvement, particularly related to usability, tailoring, and the integration of professional or peer support. In line with Lau and colleagues' meta-ethnographic review of perinatal women’s experiences with digital interventions [23]. Our findings reinforce the acceptability of digital approaches, while adding novel insights into women’s expectations of follow-up after elevated EPDS scores, the differing needs of primiparas and multiparas, and the importance of proactive rather than self-initiated help-seeking.

Women generally perceived Mamma Mia as easy to use and integrate into daily routines, describing it as simple, user-friendly, and accessible. Ease of use and usability have previously been found as valuable in other studies [23]. At the same time, participants emphasized that design features could either facilitate or hinder engagement. The lack of a mobile application, limited navigation options, and periods with weekly pacing of sessions were identified as weaknesses or threats, particularly for women balancing childcare or work responsibilities. Technology-related factors are a well-known barrier to engagement [2], and our findings suggest that flexibility and autonomy are important to maintaining engagement in digital interventions during the perinatal period. Technological enhancements such as mobile applications, adaptive pacing, and improved navigation could strengthen usability and align the intervention more closely with women’s everyday lives. The psychoeducational content of Mamma Mia was highly valued for offering timely, relevant information and practical exercises that promoted self-care and well-being. These findings align with previous research highlighting the role of psychoeducation in strengthening coping, confidence, and well-being in the perinatal period [11, 23], also regarding Mamma Mia specifically [9]. Beyond knowledge acquisition, the intervention appeared to foster emotional awareness and reassurance. Psychoeducation and repeated assessments with the EPDS, prompted women to reflect on their mood, while the program’s affirming tone normalized a range of emotional experiences. This combination of knowledge, reflective tools, and validation supported participants in understanding, navigating, and accepting emotional fluctuations. Repeated assessments were highlighted as valuable, given that mood can fluctuate throughout the day or week [1, 35]. In Norway and other countries, screening for depressive symptoms typically occurs at 6–8 weeks postpartum using pen and paper EPDS in primary care [5, 18]. Repeated digital measures may help identify depressive symptoms and changes over time, facilitating clinical assessment and early intervention [12]. However, participants suggested more interactive features and personalized feedback, which could reinforce learning and increase emotional impact.

Participants valued the credibility of information provided in the program. Lau and colleagues highlight the necessity of credible resources. Unlike previous studies that highlighted cybersecurity concerns [23], only one participant in our study expressed concerns about data sharing. This could be attributed to the information provided about data collection, trust in the developers or researchers, or the recruitment personnel. On the other side, some participants felt that writing reflections or answers to tasks was of little value since no one would read or give feedback on their input. Additional suggestions included further tailoring of sessions based on individual EPDS scores and that the program could alert healthcare providers. Some suggested that healthcare providers should proactively contact them based on their EPDS scores rather than expecting them to seek help independently.

A recurring theme was that “one size does not fit all”. Women highlighted that their needs varied depending on prior parenting experience, mental health status, and life circumstances. Multiparous women often felt that content was repetitive and less relevant, yet they identified gaps in support specific to their situation, such as preparing older siblings for a new baby or managing multiple caregiving responsibilities Experience grant multiparous women knowledge about perinatal related topics, birth, childcare, and development, and a universal intervention may provide little new knowledge. This experience may also apply also to the health care system, its pathways, and how to seek help. Similar views have previously been reported, including that multiparous women lack more in-depth information in the program [9]. Previous studies have indicated that the personalization of health apps enhances their appeal [23].

Women with elevated depressive symptoms articulated yet another set of needs. The women in this study described themselves as worried, unwell, tired, and in need of support, rather than depressed. This aligns with studies on perinatal depression, which indicate that depressive symptoms can be hard to recognize due to fear of disclosure, stigma, or shame [6, 7]. Feather and colleagues (2016) suggested that users’ psychological experiences of interventions are influenced by their symptoms and the mode of delivery [11]. This implies that women’s experiences might be shaped by both their depressive symptoms and their need for further support during the intervention. Many women identified the lack of human feedback on elevated EPDS scores as a weakness and expressed the need for additional support from health personnel. The program is designed to provide tailored information for elevated scores by encouraging women to seek social support or contact their healthcare provider. However, participants wanted feedback on how to proceed if they had elevated depressive symptoms and where to seek help and found the help-seeking process difficult. Additional suggestions for support included peer support, digital forums, recovery stories from other women, and access to mental health personnel through the program. Being able to connect with others through digital intervention can assure users, normalize experiences, and increase engagement in the intervention [2]. This further aligns with research on treatment preferences and digital interventions which found that women want professionals and peers to validate their experiences, acknowledge their needs, and provide compassionate perinatal-specific care [23, 37]. Peer support, in particular, can validate feelings, reduce isolation, and promote hope, but it must be tailored to meet women’s specific needs [37].

A systematic review highlights the importance of implementing additional interventions alongside routine care and screening to improve support for perinatal women with depressive symptoms [3]. Participants in this study expressed a desire for healthcare professionals to provide follow-up and address their diverse needs and experiences with the intervention. Guidance for multiparas and those with depressive symptoms was also suggested, emphasizing the value of self-care components and the need for supportive, non-judgmental dialogue with professionals. Participants preferred proactive contact from healthcare providers based on EPDS scores rather than initiating contact themselves. Therefore, combining Mamma Mia with routine screening and healthcare provider visits could better meet the needs of women with elevated depressive symptoms.

### Perspectives

Although the interviews in this study were conducted a decade ago, the insights remain highly relevant and have actively informed the ongoing development of Mamma Mia. Since the completion of the original randomized trial and interviews, the program has undergone continuous refinement and been redesigned into a mobile application, directly addressing participants’ suggestions for greater accessibility and usability. Key improvements include the integration of push notifications, enhanced navigation, and the addition of a self-help toolbox. Importantly, the expressed need for increased feedback and professional follow-up is now being implemented in Maternity and Child Health Services in both Norway and the USA, as part of a second randomized trial evaluating the effectiveness of guided versus unguided versions of *Mamma Mia* [21].

In Norway, the Directorate of Health holds the license for the program, and *Mamma Mia* is offered free of charge to all pregnant women, underscoring a national commitment to strengthening perinatal care through digital health interventions [27]. For users outside of Norway, the program can be entered via the Wellmo app from Appstore (Apple or Android) by register with a username and password ("To registration").

Perinatal depression remains challenging to diagnose and manage due to overlapping symptoms with normal pregnancy experiences, women’s difficulties in recognizing or disclosing distress, and stigma, guilt, or shame that hinder help-seeking. Variability in the use of screening tools and limited professional awareness further complicate early detection. To address these gaps, prevention and early intervention strategies, including stepped care and digital tools, have gained prominence. A critical challenge is to strengthen women’s willingness and ability to seek professional help while supporting healthcare providers in recognizing and responding to early symptoms [12]. The *Mamma Mia* study contributes to this field by showing how digital interventions can support prevention through psychoeducation, normalize women’s experiences, and facilitate early symptom awareness. At the same time, the findings highlight that women with elevated symptoms require more than information, they seek personalized feedback and proactive support to overcome barriers to help-seeking. By emphasizing both preventive self-care and the need for professional follow-up, *Mamma Mia* exemplifies how digital tools can complement routine perinatal care and help bridge gaps in detection, support, and timely intervention.

Looking ahead, the role of *Mamma Mia* and similar evidence-based digital interventions in perinatal care will hinge on their successful integration into routine health services. This study’s findings continue to underscore the importance of tailoring digital interventions to women’s diverse needs, as well as combining them with standardized primary care to strengthen early detection and intervention. Together with emerging evidence, these insights contribute to a broader vision of perinatal care where digital and professional support are meaningfully combined to improve maternal mental health and, ultimately, child and family outcomes.

### Strengths and limitations

Trustworthiness (i.e., credibility, dependability, and transferability) [22] was carefully considered throughout this study. Credibility was enhanced by recruiting women with elevated depressive symptoms at baseline, aligning with the study’s aim to explore experiences of women with possible depression. Diversity in age and number of children contributed to a variation in perspective. The iterative analysis process conducted both individually and collaboratively by the authors until consensus was reached, and supported by quotations, further strengthens the credibility. A notable limitation of this study is the small sample size, which is characteristic of qualitative research methodology. Even if the majority of participants had completed most sessions, one participant had only completed one session, limiting her experience with *Mamma Mia*. Additionally, those who agreed to be interviewed may have held more favorable views of *Mamma Mia*, potentially introducing bias.

Transferability is limited to the Norwegian context, characterized by universal access to maternity care, high public trust, and cultural homogeneity. These factors likely influenced participants’ perceptions, particularly regarding digital data handling and expectations of responsive care. In more diverse or resource-constrained settings, concerns about privacy, stigma, or unequal access may affect the acceptability of digital interventions. However, the Norwegian context also offers valuable insights; if women in a high-resource system still report unmet needs for personalization, relational support, and proactive follow-up, these may be universal challenges rather than context-specific ones. Dependability was strengthened by the use of a semi-structured interview guide, allowing for consistency while enabling participants to share unique perspectives and experiences with *Mamma Mia*. Finally, the authors’ preconceptions are important when reflecting on dependability. The authors’ varied backgrounds in health care and research enriched the analysis, allowing for interpretations from multiple perspectives (Additional File 1). Despite these limitations, the study’s main strength lies in its contribution to understanding how women with elevated depressive symptoms experience *Mamma Mia*, highlighting their needs for additional care and offering suggestions for improving the intervention.

### Recommendations for further research

Future research should explore how women use *Mamma Mia* in combination with support from health personnel. An examination of a guided version would provide valuable knowledge about experiences of support. Further research is thus needed to understand perinatal women’s motivation to use technologies to better support their engagement and perinatal health literacy. This understanding can inform the development of digital interventions and the integration of these interventions into perinatal women’s daily lives, self-care and health personnel’s care practices.

### Implications for policy and practice

This study highlights both strengths and areas for improvement in the current version of *Mamma Mia* offering valuable insights for future development and implementation. The participants identified several enhancements that could increase engagement and support during the transition to motherhood. Designers should consider further tailoring content that reflects individual needs and previous experiences. Future versions of the intervention should be app-based and structured with a menu of sessions to choose freely. The participants also recommended platforms for peer-to-peer support or reading about women who had experienced depression themselves. Understanding how women perceive and experience *Mamma Mia,* including how well it aligns with their expectations and personal circumstances. is essential for improving both effectiveness and adherence. Their feedback also underscores the importance of integrating digital tools with additional support pathways, such as access to health personnel and referrals to specialized care when needed. Future versions of *Mamma Mia* could include features for alerting health care providers when users report high levels of distress, enabling timely follow-up and personalized care. Such features would strengthen the link between digital interventions and routine maternity care.

From a policy perspective, universal digital interventions like Mamma Mia have the potential to reach a broad population of women at risk of developing depression. When integrated with existing health care systems, they can promote early identification, wellbeing, and tailored support, especially combined with routine care.

## Conclusion

The results of this study indicate a need to provide support and tailor follow-up of interventions in primary care. Overall, user experience is important in delivering acceptable and useful interventions. An understanding of the experiences of Mamma Mia, including expectations and fit to the individual, is essential to increasing effectiveness and adherence to the intervention and providing additional support and referrals to treatment or specialized care if needed. Combining routine care with interventions and person-centered approaches, including the use of Mamma Mia, has the potential to reduce maternal depression and alleviate the burden of depressive symptoms. The endorsed utilization of Mamma Mia can positively impact mental health, encouraging help-seeking behavior for adequate support. Integrating these strategies in primary care, alongside accessible collaboration with psychologists and referral services, holds promise for enhancing maternal care during the transition to motherhood.

## Supplementary Information

Supplementary Material 1.

## Data Availability

The datasets used and/or analyzed during the current study are available from the corresponding author on reasonable request.

## Abbreviations

COREQ: Consolidated criteria for reporting qualitative research
EPDS: The Edinburgh postnatal depression scale
GP: General practitioner
GW: Gestational week
PND: Perinatal depression
SWOT: Strengths, weaknesses, opportunities, threats
