# Supplementary material for: ’One size does not fit all’: a qualitative study of women’s experiences of a self-guided digital intervention, Mamma Mia
Source: BMC Prim Care. 2025 Dec 8;26:390. doi: 10.1186/s12875-025-03074-8 (PMC12683907; doi:10.1186/s12875-025-03074-8)
Supplement: Supplementary file 1 — Supplementary Material 1. [file 12875_2025_3074_MOESM1_ESM.docx]

# **Additional File: Analysis using the Framework Method**

A main feature of the Framework method is the matrix development: rows (codes/themes/subthemes), columns (interviewees), and cells of summarized data. This structure enables the researchers to systematically develop and reduce the data to analyze it by case and code. This provides greater transparency in the data analysis process, allows for comparisons across and within cases, and further allows us to identify similarities and differences more clearly. The Framework Method is useful for research projects with multiple researchers and to obtain a holistic and descriptive overview of the entire dataset (Gale et al., 2013). As part of a multi-disciplinary research team FD & ESO led the analysis of data to explore women’s experiences with an internet intervention, Mamma Mia, to prevent depression and promote well-being. Regular meetings facilitated the critical exploration of the data, discussion of and agreements on the interpretation of the statements. The analysis was partly done and overseen by an experienced researcher to ensure quality and rigor (FD).

In this study, we used semi-structured interviews to collect data from 12 participants (cases) from an RCT.

**Stage 1: Transcription**

To ensure similarity in transcription style across the whole dataset, one person () was responsible for carrying out transcription. The team examined transcripts to ensure comparable formatting, until satisfied that any inconsistencies had been resolved.

Transcripts were supplemented with notes made during and after interviews, as well as during familiarisation with the material.

**Stage 2: Familiarisation with the interviews**

Members (FD & ESO) of the research team thoroughly read and re-read each transcript to become familiar with the whole data set. This familiarisation process is essential when the researcher analysing the data had not been present during the interview.

**Stage 3: Coding**

One member (FD) of the research team initially coded the transcripts in NVivo. Units were coded according to themes, developed inductively from the material. Codes were named after recurring terms and meaning units. Individual quotations were reread and compared to the wording of subsequent themes and subthemes to ensure the retainment of the original meaning conveyed by the participants. Because participants provided statements in Norwegian, all themes, and subcategories, first emerged in Norwegian and were translated to English after the completion of the analysis.

| **Strengths** | **Weaknesses** |
| --- | --- |
| **Self-care and knowledge**  Reflection  Relaxation  Relevant information  Guidance and tips  **Usability**  User friendly  Flexible timing for use  Vernacular language  Research-based  Funny pictures  Presentation of content  Program download  Start after birth  Reminders, delivery, availability  Regularity and repetition  Division into rooms  **Awareness and reassurance**  Awareness of one’s wellbeing  Discussions with partner  A break in everyday life  Focusing on what is important  Care and validation  Normalization  Reminder of partner support and relationship | **One size does not fit all**  Too elementary  Too much emotionality  Did not fit my situation  Not for all  No need for help and support  **Usability**  Program use on smartphone  Boring film clips  Too much to read and time-consuming  Noone reads what I write  Information sheets  Navigation  The persons in the program  Frequent sessions  Too short sessions  EPDS concluded help-seeking  Hard to remember to use the program  To have someone to contact |
| **Opportunities** | **Threats** |
| **Usability**  Other illustrations  More options  Further reading tips  Menu  Instruction videos  More information about labour  More facts, to the point information  More content, variation and interaction  Notifications and mobile application  Reminder of the last EPDS, monitoring  Dissemination of the program  Making the information mine  **Seek more help, information and support**  Personal experiences and professional advice  Collaboration midwives, GPs, Child Health Care Services  Support in difficult situations  Guidance on help-seeking  Someone to contact | **Usability**  Use of computer  Use on smartphone  Complete sessions within intervals  Oblivion  Not tangible  Login  Self-report of emotions  Writing down and not getting responses  Making the information my own  OMG, I need to do this too  Getting started  **One size does not fit all**  If one is depressed or distressed  Universal design  Many different users, and different needs  Mostly for primiparas  **Seek more help, information and support**  Lack of partner involvement  Having someone to contact |

**Stage 4: Developing a working analytical framework.**

Codes grouped in clusters around similar and interrelated ideas in NVivo were guiding the development of the matrix. This formed the initial analytical framework.

The final framework consisted of 5 categories, each with a brief explanatory description of their meaning.

The example below shows three categories from the final analytical framework with constituent codes and descriptions of codes.

| **CODE** | **DESCRIPTION AND EXAMPLE UNIT** |
| --- | --- |
| **Usability** | |
| *User-friendly*  *Flexibility*  *Vernicular language*  *Research-based*  *Funny pictures*  *Presentation*  *Downloading*  *Mobile application*  *Navigation, menu, index*  *Reminders*  *Availability*  *Different rooms*  *Scrolling* | Usability (see [ISO 9241-11](https://www.iso.org/obp/ui/#iso:std:iso:9241:-11:en)) is the extent to which a product can be used by specified users to achieve specified goals with effectiveness, efficiency, and satisfaction in a specified context of use. Effectiveness is fundamental as it is about achieving the intended goal(s). Efficiency is about the resources (such as time or effort) needed by users to achieve their goals so it can be important. In addition, it is important that users are satisfied with their experience, particularly where users have discretion over whether to use a product and can readily choose some alternative means of achieving their goals. In this part of [ISO/TS 20282](https://www.iso.org/obp/ui/#iso:std:iso:pas:20282:en), accessibility is operationalized as the extent to which a product can be used with effectiveness, efficiency, and satisfaction by people from a population with the widest range of characteristics and capabilities to achieve a specified goal in a specified context of use <https://www.iso.org/obp/ui/#iso:std:iso:ts:20282:-2:ed-2:v1:en>  «Should be easy to manoeuvre, and you have your phone with you. Because I noticed that they go on, log back into the Mamma Mia program and find my way around and which was it, it was much heavier Having a menu of exercises and conversations, makes it much easier to find your way around.» |
| **Seek more information, help and support** | |
| *Personal experiences from other women with depression*  *Feedback from health personnel*  *Collaboration with midwives, GPs, PHNs*  *Support in difficult situations*  *Guidance on help-seeking*  *Someone to contact*  *Lack of partner involvement* | Individualized follow-up, needs-oriented support from a professional  Face-to-face interaction and support  Mamma Mia alerting health personell when scores on the EPDS are above cut-off or with a chat function  Guidance on material and themes  Health personell |
| **One size does not fit all** | |
| *If one is depressed*  *Reaching out to all pregnant women*  *Different groups, different needs*  *Mostly for primiparas*  *Universal intervention not sufficient when depressed*  *Reaching out to all pregnant women*  *Too elementary for multiparous women* | A universal intervention does not meet all needs.  Multiparous women want additional modules for preparing siblings. |

**Stage 5: Applying the analytical framework.**

Themes and subthemes initially developed in NVivo formed the analytical framework in Microsoft Excel, codes were reduced, and themes and subthemes were reviewed, according to answering research questions.

**Stage 6: Charting data into the framework matrix**

The matrix comprised one row per theme/subcategory and one column per participant. We (ESO) abstracted data from transcripts in NVivo for each participant and theme/subtheme, summarised it using verbatim words and inserted it into the corresponding cell in the matrix. References to potentially interesting quotations were inserted in a separate sheet. FD oversaw and reviewed the matrix.

**Stage 7: Interpreting the data.**

Themes were generated from the data set by reviewing the clusters of codes and their meaning.

| **Example of memo** | |
| --- | --- |
| *Theme: Awareness*  The quality or state of being aware, consciousness; (also) the condition of being aware (of something or that something is) (Oxford English Dictionary) | A Just one of the participants addressed being depressed as a reason why additional support was needed.  Transitions theory describes the need to be connected as a way of facilitating transitions, this may be expressed by both the suggestion to include other women’s experiences and that Mamma Mia can provide contact with health personnel or that healthcare providers in the Maternity and Child Services should know about Mamma Mia and provide guidance on material and discussion about topics in Mamma Mia.  Why aren’t midwives and PHNs mentioned more frequently as support? Reduced number of consultations during pregnancy, midwives and PHNs must introduce both mental health and interventions to be a service to contact for mental health issues, lack of support, or in general questions about health. Barriers to help-seeking: stigma, guilt, lack of relations and trust in health personnel, it is normal to feel this bad and one should be able to cope alone (Morrell).  Normalising: their experience is not uncommon and other women got through it.  Legitimate to make time for themselves.  Several participants reported that the EPDS was beneficial and that the recurring EPDS was useful. Some of the participants reported that the EPDS should be improved to provide a report on the previous EPDS scores, remind and alert women if symptoms increase or to monitor one’s responses. |
|  |  |

| **Transcripts** | | |
| --- | --- | --- |
| Det er jo veldig lett og enkelt å bruke. Jeg har det på telefonen og det kommer opp en mail, så det er veldig brukervennlig. Jeg synes det var fine lengder på ting, at det tok passelig tid. | *After all, it is very straightforward and easy to use. I use Mamma Mia on my phone, and an email comes up, so it is very user friendly. I think that each session took a reasonable amount of time.* | P06, Strength |
| Og så har jo jeg oftest lest, sittet og hørt på det på toget, på iPhone… Når en skal fylle ut alle disse spørsmålene, så er det jo litt klønete på iPhone. Så det kunne jo vært et forbedringsområde. At du kunne fått det mer mobiltilpasset. | *And then I have mostly read and listened to the sessions on the train, on my iPhone… Answering all these questions, it is a bit clumsy on the iPhone. That could have been an area of improvement. It could be more mobile-friendly.* | P04, Opportunity |
| …så må man klikke seg gjennom en og en slide, eller side, for å komme seg videre. Jeg skulle ønske noen ganger at det var en sånn toppmeny hvor du bare kunne klikke... Og det å kunne manøvrere litt lettere inne på selve programmet, det har jeg savna. | *… you have to click through one slide, or page, to move on. I wish sometimes there was a top menu like that where you could just click… Being able to maneuver a little bit easier in the program, that’s what I have missed.* | P01, Opportunity |
| De filmene og sånn har ikke vært helt aktuelle for meg da. Sånn at de har jo jeg da bare hoppet over, når jeg har skjønt tema… Det måtte vært at man kunne ha en sånn ‘Hoppe over dette kapittelet’-knappfor min del. | *Those movies have not been quite relevant to me then. Therefore, I have just skipped them when I have figured out the topic…It would have to be that you could have a 'Skip this session' button for me.* | P05, Opportunity |
| Jeg ønsket meg lenger intervaller mellom hver, ikke én oppgave eller én e-post hver uke, jeg ville gjerne hatt i alle fall to uker mellom. Så eventuelt om det hadde vært mulig å få lov til å velge selv. | *I wanted longer intervals between each session, not one assignment or one email each week. I would have liked to have at least two weeks in between… Therefore, if it had been possible to be allowed to choose for oneself…* | P09, Opportunity |
| Så det eneste er at jeg følte at du hang bak på, det er mange ting du skulle gjøre hele tiden, og så hang du bakpå med dette òg, så det ga en veldig dårlig følelse. | *The only thing is, I felt like I was lagging behind, there's a lot of things you were supposed to do all the time, and then you were lagging behind with this as well. That was a truly bad feeling.* | P09, Weakness |
| Det jeg synes har vært godt med det, har vært de små rådene som har kommet underveis. Ikke nødvendigvis alle de filmsnuttene, men de små rådene, både i parrelasjon og i relasjon med barnet. Det er veldig grei tå ha et program som følger utvikling. | *What has been good about it, has been the small pieces of advice that have been provided. Not all of the film clips, but the small advice regarding both us as a couple and the relationship to the child. It is truly good to have a program that follows [pregnancy and the child's] development."* | P02, Strength |
| Alle har gode dager og dårlige dager, så hvis jeg synes at en dag har vært veldig trist, at det å finne en positiv ting i løpet av dagen gjorde at det ble litt mer positivt. | *Everyone has good days and bad days. So, if I feel that one day has been truly sad, finding a positive thing during the day made it slightly more positive.* | P02, Strength |
| nå har ikke jeg lagt på meg så mye, så var jordmor veldig bekymret for det, så da brukte vi hele timen på egentlig å ringe til A-hus, bare for å få en ekstra ultralydtime, og da var det sånn, ja, da var vi ferdig, og så satt jeg egentlig og hadde litt sånn, okei, nå skal jeg snart føde, termin begynner å nærme seg, og permisjonen begynner å nærme seg med stormskritt nå, og jeg bare kjente at jeg hadde fullt av tanker og var bare sånn, å, herregud, nå tror jeg faktisk jeg kapitulerer, orker ikke mer nå. Så kom den e-mailen i går, og den der avslapningsgreien og puste og sånne ting, og da kjente jeg liksom okei, nå kan jeg takle det, det går bra liksom. Så det har jeg likt veldig godt. | *I had not put on that much weight, so the midwife was truly worried about it. We spent the whole consultation calling the hospital, just to get an extra ultrasound appointment, and then it was like, yes, then we were done, and then I was ‘okay, I'm going to give birth soon. It is getting closer, and my maternal leave is approaching fast’, and I just felt like I was full of thoughts and was just like, ‘Oh, my God, now I actually think I'm capitulating, cannot take it anymore now’. Then, that email came in yesterday and the session with the relaxation and breathing exercise and stuff like that, and then I kind of felt ‘Okay, now I can handle it, it is okay somehow’. I have truly liked that.* | P10, Strength |
| Og, jeg vet ikke helt hvordan det har funket i forhold til meg selv, det har jo funket på den måten at jeg har sluttet å røyke i graviditeten, for babyens skyld. | *I'm not sure how it is worked out concerning myself; but it worked out the way that I have stopped smoking during pregnancy, for the baby's sake.* | P04, Strength |
| Jeg har hatt utfordring med hvordan kommuniserer man til en toåring at det er en baby i magen til mamma’, ikke sant? Sikkert mye som foregår hos han. Så litt råd og veiledning rundt det kunne jo vært perfekt, for eksempel. | *I have had the challenge of how do you communicate to a two-year-old that ‘there's a baby in my mommy's womb’, right? Probably a lot going on with him. So a little advice and guidance about that could be perfect, for example.* | P08, Opportunity |
| Man er veldig trøtt og skal man ta hånd om seg selv og at hånd om en seksåring, det har vært en utfordring som jeg da ikke har fått noe hjelp om i Mamma Mia. | *You're very tired, and going to take care of yourself and take care of a six-year-old, that has been a challenge that I have not received any help with in Mamma Mia.* | P07, Threat |
| Fordi at jeg er av dem som sikkert hadde hatt veldig godt av å ha det programmet første gangen, fordi jeg fikk jo fødselsdepresjon da. Og jeg var veldig uforberedt. | *Because I'm one of those who probably would have benefited from having that program the first time because I got postpartum depression then. And I was very unprepared.* | P12 Strength |
| Kanskje man skulle ha differensiert på de som sier de har …, de som man føler strever da… Det er sikkert større behov for de som trenger den støtten også. Det er dumt å ha det forskjellig for dem også. | *Perhaps one should have differentiated between those who say they have..., those who feel they are struggling then... There is certainly a greater need for those who need that support as well. But, it is silly to make it different for them too.* | P05, Opportunity |
| Jeg synes det har vært greit å ha Mamma Mia, fordi det har minnet meg litt om hva det er jeg går gjennom. Selv om jeg på en måte visste det. Så er det litt, som jeg sa da, den tiden til å tenke, ja, det er faktisk et barn inni her Ja, det er litt travelt. Absolutt. Så du får litt tilbake den undringen og gleden ved å være gravid. et pusterom i hverdagen, ja, det synes jeg det var. | *I think it is good to have Mamma Mia because it reminded me of what I was going through. Even though I kind of knew it... have the time to think, and yes… “There is a child in here”. Yes, it is a busy time. Absolutely. To get into wonder and joy during pregnancy. A breathing space in everyday life, yes, that is what I think it was.* | P06, Strength |
| Jeg synes også de har vært bra disse spørsmålene om hvordan det går, om man er deprimert eller ikke. Og det har jeg syntes har vært bra, for meg også. Det har fått meg til å tenke litt på hvordan jeg har det egentlig. Og da har jeg også tenkt lite granne på hvorfor har jeg det sånn. Og så at det kommer igjen. Kanskje når det gjelder de spørsmålene, at man kunne få påminnelse om hva man svarte sist. | *I also think they have been good these questions about how you're doing, whether you're depressed or not. I think that has been good, for me, too. It made me think a little bit about how I'm truly feeling. I have also thought about why I feel that way. And the repetition [the EPDS]. Perhaps in the case of those questions, that one could be reminded of what one answered the last time.* | P07, Opportunity |
| Vi fikk jo snakket litt mer om det, om hvordan vi gjør ting og så sette av tid til å prate sammen, og om familieting ikke bare den hva som skal gjøres denne uka. Litt om hvordan vi synes vi har det egentlig, å tenke litt gjennom det. Om det er noen situasjoner vi har vært oppi som vi ønsker vi hadde gjort annerledes. Det har gjort det litt lettere å ta opp ting, noe man tenker på som plager en. Ja, ta opp ting på en mer konstruktiv og rolig måte, i stedet for å vente til det koker over. | *We [the participant and partner] got to talk a little bit more about it, about how we do things and then set aside time to talk, and about family things, not just what we have to do this week. A little bit about how we think we're truly doing, to think it through. If there were any situations, that we wished we had dealt with differently. It has made it easier to bring things up, issues that are bothering us. Yes, bring subjects up more constructively and calmly, rather than waiting until it boils over.* | P06, Strength |
| Ja, jeg vet ikke helt hvordan, men på en eller annen måte så har det føltes positivt. jeg hadde større behov for å føle at det fortsatt er meg oppi alt det. At jeg får lov til å ha alle disse følelsene og får lov til å være meg selv oppi det. Fordi at jeg ikke bare har følt at dette fantastiske familielivet skjer. Det er liksom noe jeg har måttet vokse til. Og det har jeg. Gjennom graviditeten. Sånn at den inndelingen har vel kanskje gjort at jeg har fått lov til å være meg selv... jeg har rett til å føle det jeg føler. Samtidig som kanskje det har gjort at jeg har glidd litt lettere inn i den rollen som jeg er i nå. | *Yes, I'm not sure how, but it somehow felt positive. I had a greater need to feel like it was still me in all that. That I'm allowed to have all these feelings and be allowed to be myself in it. Because I have not just felt this wonderful family life happening. It is kind of something I have had to grow into. And I have. Through pregnancy. The division [*into rooms*] has probably allowed me to be myself… I have the right to feel what I feel. At the same time, maybe it made me merge a little easier into the role I'm in now.* | (P04, Strength) |
| På en annen måte så tenkte jeg når jeg begynte med Mamma Mia-programmet, at det kommer til å bli bra, fordi at hvis jeg blir deprimert og hvis jeg føler meg veldig dårlig, så kommer jeg til å bli fanget opp. Men det blir man ikke… Så jeg vet ikke om man på en eller annen måte kan bli fanget opp, om det er virkelig ikke går bra for en… Jeg hadde helst villet at noen hadde spurt meg er det okei at vi ringer. | *On a different note, I thought when I started the Mamma Mia program, it is going to be good, because if I get depressed and if I feel really bad, I'm going to get identified. However, you will not… So, I do not know if one can somehow get identified if it is truly not going well for one… I'd rather had someone ask me, is it okay for us to call you?* | P07, Weakness |
| Jeg har savnet en person å diskutere ting med, om det som programmet tar opp, så hadde jeg heller villet ha en person å diskutere med. Men programmet er jo et internettbasert selvhjelpsprogram, så det er jo ikke en person som man skal prate med. Jeg hadde nok trengt å diskutere hvordan det går med meg, fordi at jeg har vært… deprimert lenge, tatt antidepressiva. Det jeg virkelig trengte, var noen å diskutere det med, og noen som jeg kunne si at jeg har det slik og føler det slik og jeg er så trøtt. Og noen som da kunne gi personlig respons på det, og si ja, men det er sånn det er. Eller si at … enten si at ja, men det er helt normalt og det er slik det er, og du reagerer helt normalt slik alle andre, eller noen som kan si at dette ser ikke så bra ut, kanskje du begynner å bli litt deprimert, du burde nok følge det opp litt mer. Det hadde jeg trengt. | *I have missed a person to discuss things with, about what the program discusses, I'd rather have a person to discuss with. But it is a internet based self-help program, it’s not a person to talk to. I probably would have needed to discuss how I'm doing, because I have been... depressed for a long time, taking antidepressants. What I needed was someone to discuss it with, and someone who I could say that ‘I am like this and feel that way and I'm so tired’. In addition, someone who could then personally respond to it, and say yes, but that is the way it is. Or say that... Either say that yes, but it is perfectly normal and that is the way it is, and you react perfectly normal like everyone else, or someone who might say this does not look so good, maybe you're starting to get a little depressed, you should probably follow it up a bit more. I needed that.* | P07, Weakness |
| … få til et samarbeid med enten fastlege eller helsestasjon og jordmortjenesten. Det var tilfeldig at jeg kom over dere igjennom en nettside. hvis man følger opp og sånn, så er det nok veldig mange som kan dra nytte av det. | *…establish collaboration with either a GP or a health center and the midwifery service. It was a coincidence that I came across Mamma Mia through a website. …there are probably many people who can benefit from it.”* | P04, Opportunity |
| Det hadde vært greit om man fikk en slags bekreftelse, for jeg tenker at hvis man sitter og føler at man ikke har det helt topp, at det kan være litt vanskelig å sette fingeren på hvordan man har det kanskje, og litt sånn vanskelig å erkjenne det ovenfor seg selv og andre og sånn, så i hvert fall føler jeg da i slike situasjoner enten behov for å få en fagpersons mening om saken da, på en måte si at dette tilsier at du kan være deprimert osv. At man på en måte ikke går og tenker at man sikkert, at det er meningen at man skal tåle det man går gjennom osv. få en slags bekreftelse på at du har det sånn og sånn, og eventuelt hva man kan gjøre med saken. | *It would be nice if you got some kind of validation… Because I think that if you sit and feel that you are not feeling great, that it can be a little difficult to put your finger on how you feel perhaps, and a little bit difficult to acknowledge it to yourself and others… then at least in such situations I can eighter feel the need to get a professional's opinion on the matter then, kind of say that this indicates that you might be depressed, etc. That in way you don’t go thinking… you're supposed to endure what you're going through, etc. Get some kind of confirmation that you feel this way and that, and possibly what you can do about it.* | P11, Opportunity |
| Videoer eller skriv fra av personlige erfaringer av folk som har fødselsdepresjon, hvordan føles det, osv. og litt sånn personlig fra folk kanskje, videoer eller tekststykker. Det kunne vært en fin sånn sak som man kan se på. | *Videos or texts about personal experiences with postpartum depression, how it feels, and so on... It could be a nice thing to look at...*  *Possibly some forum, a platform with others. In addition, maybe experts, a psychologist.* | P11, Opportunity |

Gale, N. K., Heath, G., Cameron, E., Rashid, S., & Redwood, S. (2013). Using the framework method for the analysis of qualitative data in multi-disciplinary health research. *BMC Medical Research Methodology*, *13*(1), 117. <https://doi.org/10.1186/1471-2288-13-117>
